# Microbial Interactions Within Multiple-Strain Biological Control Agents Impact Soil-Borne Plant Disease

**DOI:** 10.3389/fmicb.2020.585404

**Published:** 2020-10-09

**Authors:** Ben Niu, Weixiong Wang, Zhibo Yuan, Ronald R. Sederoff, Heike Sederoff, Vincent L. Chiang, Rainer Borriss

**Affiliations:** ^1^State Key Laboratory of Tree Genetics and Breeding, Northeast Forestry University, Harbin, China; ^2^College of Life Science, Northeast Forestry University, Harbin, China; ^3^Forest Biotechnology Group, Department of Forestry and Environmental Resources, North Carolina State University, Raleigh, NC, United States; ^4^Department of Plant and Microbial Biology, North Carolina State University, Raleigh, NC, United States; ^5^Institute of Biology, Humboldt University of Berlin, Berlin, Germany; ^6^Institute of Marine Biotechnology e.V. (IMaB), Greifswald, Germany

**Keywords:** microbial interaction, biological control agents, soil-borne disease, consortia, microbiome and community

## Abstract

Major losses of crop yield and quality caused by soil-borne plant diseases have long threatened the ecology and economy of agriculture and forestry. Biological control using beneficial microorganisms has become more popular for management of soil-borne pathogens as an environmentally friendly method for protecting plants. Two major barriers limiting the disease-suppressive functions of biocontrol microbes are inadequate colonization of hosts and inefficient inhibition of soil-borne pathogen growth, due to biotic and abiotic factors acting in complex rhizosphere environments. Use of a consortium of microbial strains with disease inhibitory activity may improve the biocontrol efficacy of the disease-inhibiting microbes. The mechanisms of biological control are not fully understood. In this review, we focus on bacterial and fungal biocontrol agents to summarize the current state of the use of single strain and multi-strain biological control consortia in the management of soil-borne diseases. We discuss potential mechanisms used by microbial components to improve the disease suppressing efficacy. We emphasize the interaction-related factors to be considered when constructing multiple-strain biological control consortia and propose a workflow for assembling them by applying a reductionist synthetic community approach.

## Introduction

The interest in control of plant diseases by beneficial microbes, has increased recently due to the global need for environmentally friendly alternatives to chemical pesticides and fertilizers (Handelsman and Stabb, 1996; Fira et al., 2018; Syed Ab Rahman et al., 2018). A large number of bacterial and fungal strains, as well as viruses, nematodes, and insects have been employed as biological control agents (BCAs) in the management of soil-borne crop pathogens for decades. BCAs have become a crucial component of sustainable agriculture and forestry (Cazorla and Mercado-Blanco, 2016; Alvarez and Biosca, 2017; Busby et al., 2017; Umesha et al., 2018). Although numerous beneficial microbial strains performed well against pathogens under controlled conditions in the laboratory or the greenhouse, examples of successful BCA application in commercial field-based crop production are rare (Xu et al., 2011; Mazzola and Freilich, 2017). This is mainly due to inadequate colonization of host rhizosphere connected with inefficient inhibition of soil-borne pathogen growth (Sarma et al., 2015; Mazzola and Freilich, 2017).

Different BCA consortia, consisting of two or more microbial strains [multi-strain biological control agents (MSBCAs)], are assembled to improve the stability and efficiency of disease-inhibition (Sarma et al., 2015; Mazzola and Freilich, 2017; Vorholt et al., 2017; Woo and Pepe, 2018). The biotechnological potential of microbial consortia was reviewed recently and examples for their possible applications in areas of biopolymers, bioenergy, biochemicals, and bioremediation have been presented (Bhatia et al., 2018). Here, we focus on the application of MSBCAs in sustainable agriculture. In several cases, superior disease suppression exerted by MSBCAs has been reported (Table 1). Diverse modes of action were proposed: (i) diversity in biocontrol mechanisms offered by each microbial component (Pierson and Weller, 1994; Sarma et al., 2015), (ii) occupation of distinctive niches by probiotic microorganisms resulting in more stable communities (Pierson and Weller, 1994; Pliego et al., 2008; Thomloudi et al., 2019), (iii) enhanced modulation of genetic elements acting in the community (Lutz et al., 2004), and (iv) a broader spectrum of targeted phytopathogens (Sarma et al., 2015; Thomloudi et al., 2019) may contribute to greater biocontrol activity in communities. However, our understanding of the mechanisms underlying the reinforcement of their disease-inhibitory effects by MSBCAs is still very limited.

**TABLE 1:** List of multiple-strain biological control agents (MSBCAs) against soil-borne pathogens.

**Number**	**Multiple-strain biological control agents**	**Mode of application**	**Disease**	**Pathogens**	**Potential mode of action**	**Host**	**References**
1	*Trichoderma harzianum* CECT 2413 and *Streptomyces rochei* Ziyani	Soil inoculation	Root rot	*Phytophthora capsici*	Disintegration of the hyphae and production of 1-propanone, 1-(4-chlorophenyl)	Pepper	Ezziyyani et al., 2007
2	*Bacillus cereus* AR156, *Bacillus subtilis* SM21 and *Serratia* sp. XY21	Seedling treatment	*Phytophthora* blight	*Phytophthora capsici*	Alternation of the soil bacterial community	Sweet pepper	Zhang L.-N. et al., 2019
3	*Pseudomonas aeruginosa* MBAA1, *Bacillus cereus* MBAA2 and *Bacillus amyloliquefaciens* MBAA3	Seed bacterization	Stem rot and charcoal rot	*Sclerotinia sclerotiorum* and *Macrophomina phaseolina*	Production of ammonia, siderophore and enzymes like β-1,3 glucanase, chitinase and cellulase	Soybean	Thakkar and Saraf, 2014
4	*Pseudomonas aeruginosa* PJHU15, *Trichoderma harzianum* TNHU27 and *Bacillus subtilis* BHHU100	Seed coating	White rot	*Sclerotinia sclerotiorum*	Induced systemic resistance and enhanced oxygen species managenment	Pea	Jain et al., 2015
5	*Pseudomonas* sp. S1, *Bacillus* sp. S2, *Azotobacter* sp. S3, *Azospirillum* sp. S4 and *Pseudomonas fluorescens* S5	Seedling treatment	Vascular wilt	*Fusarium oxysporum* f. sp. *lycopersici*	Induced systemic resistance	Tomato	Kannan and Sureendar, 2009
6	*Glomus intraradices*, *Pseudomonas* sp. and *Trichoderma harzianum*	Seed soaking	*Fusarium* wilt	*Fusarium oxysporum* f. sp. *lycopersici*	Production of siderophore and rhamnolipid	Tomato	Srivastava et al., 2010
7	*Bacillus subtilis* S2BC-1 and *Bacillus subtilis* GIBC-Jamog	Seed bacterization and soil application	Vascular wilt	*Fusarium oxysporum* f. sp. *lycospersici*	Direct biocontrol and induced systemic resistance	Tomato	Shanmugam and Kanoujia, 2011
8	*Trichoderma* sp. NRCB3 and *Trichoderma asperellum* Prr2	Soil inoculation and root treatment	*Fusarium* wilt	*Fusarium oxysporum* f. sp. *cubense*	Inhibition of spore germination and mycelial growth due to antibiosis and antifungal metabolites production	Banana	Thangavelu and Gopi, 2015b
9	*Bacillus subtilis* GB03, *Bacillus amyloliquefacien* IN937a and *Pseudomonas fluorescens* CECT 5398	Media inoculation and seed drenching	*Fusarium* wilt and *Rhizoctonia* damping off	*Fusarium oxysporum* f. sp. *radicis-lycopersici* and *Rhizoctonia solani*	Production of siderophores and induced systemic resistance	Pepper and tomato	Domenech et al., 2006
10	*Bacillus* sp. EPB10, *Bacillus* sp. EPB56 and *Pseudomonas fluorescens* Pf1	Root soaking	*Fusarium* wilt	*Fusarium oxysporum* f. sp. *cubense*	Enhancement of the expression of defense related enzymes	Banana	Mathiyazhagan et al., 2014
11	Mixture of uncultivated endophytes derived from healthy banana plants	Root drenching	*Fusarium* wilt	*Fusarium oxysporum* f. sp. *cubense*	Antagonism and induction of the activities of host defense-related enzymes	Banana	Lian et al., 2009
12	*Glomus mosseae*, *Trichoderma harzianum* and *Pseudomonas fluorescens*	Soil inoculation	*Fusarium* wilt	*Fusarium oxysporum* f. sp. *cubense*	Physical modifications in the cell wall, growth promotion and induction of disease resistance	Banana	Mohandas et al., 2010
13	*Pseudomonas putida* C4r4, *Pseudomonas putida* Jrb2, *Bacillus cereus* Jrb1, *Bacillus cereus* Jrb5, *Bacillus flexus* Tvpr1, *Achromobacter* spp. Gcr1 and *Rhizobium* spp. Lpr2	Root dipping and soil application	*Fusarium* wilt	*Fusarium oxysporum* f. sp. *cubense*	Production of siderophores, protease enzymes, chitinase and hydrogen cyanide	Banana	Thangavelu and Gopi, 2015a
14	*Bacillus subtilis* EPB56, *Bacillus subtilis* EPB10 and *Pseudomonas fluorescens* Pf1	Root soaking	*Fusarium* wilt	*Fusarium oxysporum* f. sp. *cubense*	Increasement of the activity of defense enzymes	Banana	Kavino and Manoranjitham, 2017
15	*Pseudomonas aeruginosa* DRB1 and *Trichoderma harzianum* CBF2	Soil inoculation	*Fusarium* wilt	*Fusarium oxysporum* f. sp. *cubense*	Production of 2,4-diacetylphloroglucinol and chitinase	Banana	Wong et al., 2019
16	*Pseudomonas* sp. UPMP3 and *Burkholderia* sp. UPMB3	Soil drenching	*Fusarium* Wilt	*Fusarium oxysporum*	Increase of resistance-related enzymes, lignithioglycolic acid and pathogenesis-related proteins	Banana	Mohd Fishal et al., 2010
17	*Bacillus subtilis* GBO3, *Bacillus subtilis* MBI600 and *Rhizobium tropici*	Seed application	Root rot	*Fusarium oxysporum*, *Fusarium solani f. sp. phaseoli* and *Rhizoctonia solani*	Production of siderophores	Dry bean	Estevez de Jensen et al., 2002
18	*Pseudomonas fluorescens* LPK2, *Sinorhizobium fredii* KCC5 and *Azotobacter chroococcum* AZK2	Seed bacterization	Fusarial wilt	*Fusarium udum*	Production of metabolites against the conidial germination and germ tube growth	Pigeon pea	Choure et al., 2012
19	*Stenotrophomonas maltophilia* AA1, *Ochrobactrum pituitosum* AA2, *Curtobacterium pusillum* AA3, *Enterobacter ludwigii* AA4, *Chryseobacterium indologenes* AA5, *Herbaspirillum frisingense* AA6 and *Pseudomonas putida* AA7	Seed soaking	Seedling blight	*Fusarium verticillioides*	Inhibiting fungal colonization and arresting hyphal expansion growth	Maize	Niu et al., 2017
20	*Xanthobacter agilis*, *Microbacterium* sp., *Paracoccus denitrificans*, two Enteric bacterium strains and five Coryneform bacterium strains	Seed soaking	*Pythium* damping-off	*Pythium ultimum*	Fatty acid metabolism	Cotton	McKellar and Nelson, 2003
21	*Trichoderma viride* and *Streptomyces* sp.	Media inoculation	Sudden wilting	*Pythium aphanidermatum*	-	Poinsettia	Bolton, 1980
22	Chitinophaga sp. 94, and *Flavobacterium* sp. 98	Root drenching	Damping off	*Rhizoctonia solani*	A NRPS-PKS gene cluster from *Flavobacterium* was essential for disease suppression	Sugar beet	Carrión et al., 2019
23	*Streptomyces atrovirens* N23 and *Trichoderma lixii* NAIMCC-F-01760	Soil inoculation and root treatment	Root rot	*Rhizoctonia solani*	Activation of plant defense	Tomato	Solanki et al., 2019
24	*Trichoderma virens* GI006 and *Bacillus velezensis* Bs006	Soil inoculation	Fusarium wilt	*Fusarium oxysporum* f. sp. *phaseoli*	Formation of biofilms and production of antimicrobial compounds	Cape gooseberry	Izquierdo-García et al., 2020
25	*Bacillus cereus* AR156, *Bacillus subtilis* SM21 and *Serratia* sp. XY21	Seedling and soil drenching	Verticillium wilt	*Verticillium dahliae*	Induced systematic Resistance and secretion of anti-fungal metabolites	Cotton	Yang et al., 2014
26	*Pseudomonas* sp. CHA0, *Pseudomonas* sp.PF5, *Pseudomonas* sp.Q2-87, *Pseudomonas* sp.Q8R1-96, *Pseudomonas* sp.1M1-96, *Pseudomonas* sp. MVP1-4, *Pseudomonas* sp.F113, and *Pseudomonas* sp. Phl1C2	Root drenching	Bacterial wilt	*Ralstonia solanacearum*	Competition for resources and interference with the pathogen	Tomato	Hu et al., 2016
27	*Ralstonia* spp. QL-A2, *Ralstonia* spp. QL-A3, *Ralstonia* spp. QL-A6, *Ralstonia* spp. QL-117 and *Ralstonia* spp. QL-140	Root drenching	Bacterial wilt	*Ralstonia solanacearum*	Resource competition	Tomato	Wei et al., 2015
28	*Serratia plymuthica* A294, *Enterobacter amnigenus* A167, *Rahnella aquatilis* H145, *Serratia rubidaea* H440, and *S. rubidaea* H469	Tuber soaking	Potato soft rot	*Pectobacterium spp., Dickeya spp.*	Production of antibiotic compounds, biosurfactants and siderophores	potato	Maciag et al., 2020
29	Tomato rhizosphere microbiome	Transplantation	Bacterial wilt	*Ralstonia solanacearum*	*Flavobacteriaceae* sp. TRM1 could suppress *Ralstonia solanacearum* disease development	Tomato	Kwak et al., 2018
30	Eggplant and cucumber rhizosphere microbiome	Root drenching	Root knot	*Meloidogyne* spp.	Direct antagonism and/or induction of plant resistance	Tomato	Zhou et al., 2019
31	Root associated synthetic multikingdom assemblages	Soil inoculation	−	Fungal pathogens	Bacterial microbiota suppresses fungal pathogens	Arabidopsis	Durán et al., 2018

Interactions in communities of plant-associated microbes are essential for plant health (Whipps, 2001; Frey-Klett et al., 2011; Kemen, 2014; Hassani et al., 2018). A well-known example is disease suppressive soil. They are defined by their ability to suppress plant diseases such as “take-all” disease in wheat caused by the fungal pathogen *Gaeumannomyces graminis*. The suppressive effect is due to the presence of 2,4-diacetylphloroglucinol produced by a group of soil-borne *Pseudomonas* spp. (Kwak and Weller, 2013).

The interplay among the members of MSBCAs might be relevant to their elevated disease-suppressing effect. It is necessary to pay attention to the microbe−microbe interplay-related elements when constructing MSBCAs because microbial interactions within the plant microbiome are important selective forces forming complex microbial assemblages (Hassani et al., 2018). In general, two different methods can be distinguished when BCA consortia are prepared: (i) mixing existing single-strain biological control agents (SSBCAs) according to empirical experience or (ii) preparing MSBCAs as a reductionist synthetic community (RSC) (Liu et al., 2019). In the RSC approach, defined synthetic communities (SynCom) are assembled using a limited number of isolates from the natural microbiome. In the following we prefer to use the term “SynCom” given that synthetic communities contain usually a limited number of isolates.

In this review, we provide a brief overview of the current state of the use of MSBCAs in the management of soil-borne diseases and describe potential mechanisms used by their microbial components to improve disease-suppression. We describe interaction-related factors to be considered when constructing MSBCAs and propose a workflow for assembling them as a reductionist synthetic community (Vorholt et al., 2017; Liu et al., 2019).

## Utilization of MSBCAs in Management of Soil-Borne Diseases

Selection of novel biocontrol microbial strains via isolation and screening is a permanent approach to improve the disease-controlling efficiency of BCAs. Although novel disease-suppressive strains might overcome inadequate colonization of the host rhizosphere and inefficient inhibition of soil-borne pathogen growth, the discovery of taxonomically novel isolates possessing biological disease control activity becomes more difficult over time even after extensive searches. Another promising approach, exploiting genetically modified microbial strains with improved antagonistic function has been restricted or prohibited worldwide (Migheli, 2001; Stemke, 2004). When applying BCAs in natural settings, BCAs do not act independent of their environment but interact with many indigenous microbes to become components of local microbial communities. The members of such consortia may evolve niche−specific microbial interactions to influence plant health (Whipps, 2001). There is growing interest in the use of disease-suppressing microbial communities, specifically MSBCAs, for controlling soil-borne pathogens.

**FIGURE 1:**
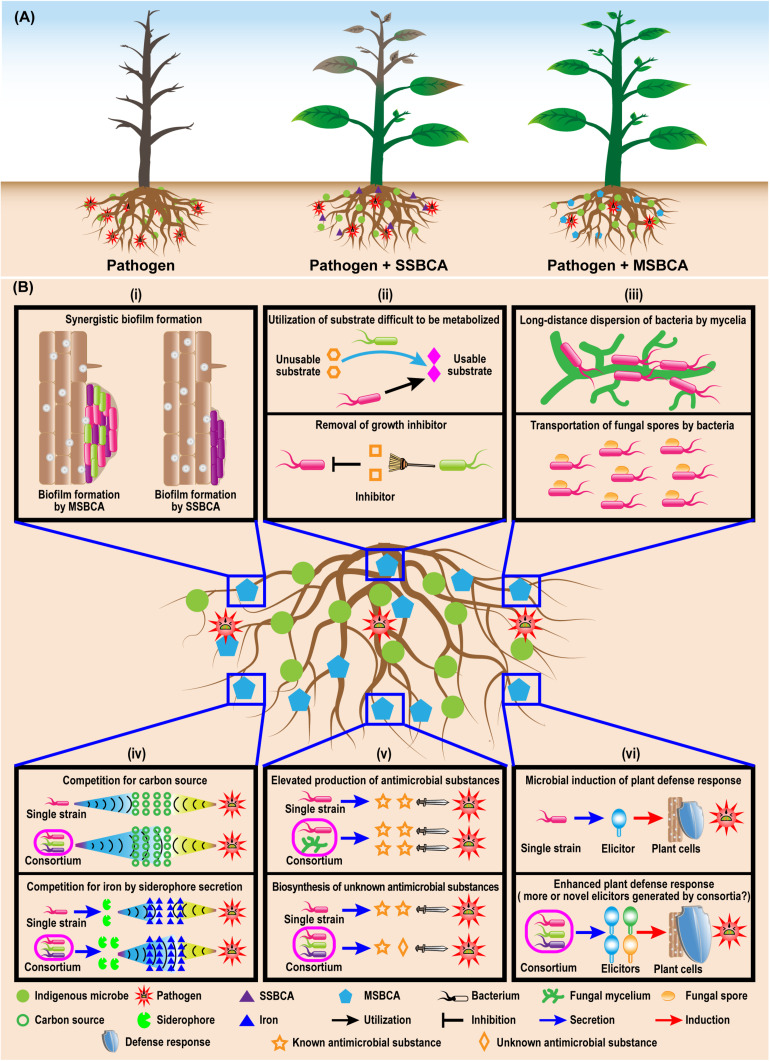
Enhanced biocontrol effects of multiple-strain biological control agents (MSBCA) against soil-borne pathogen **(A)** and the potential mechanisms underlying the elevated disease-suppressive efficacy **(B)**. (i) enhanced biofilm formation, (ii) syntrophic microbial growth promotion, (iii) facilitated migration, (iv) boosted competition for resources, (v) stimulated antimicrobial substance biosynthesis, and (vi) elevated plant defense response induction.

Multi-strain biological control agents have successfully controlled soil-borne diseases of valuable crops caused by fungi, oomycetes, bacteria and nematodes (Table 1). Several microbial combinations are possible, such as fungus to fungus, fungus to bacterium, and bacterium to bacterium. Similar to the single-strain biological control agents (SSBCAs), MSBCAs employ diverse modes of action for control, e.g., competition for resources and niches (McKellar and Nelson, 2003; Wei et al., 2015; Hu et al., 2016), production of antimicrobial compounds (Thakkar and Saraf, 2014; Santhanam et al., 2019), induction of systemic resistance (Sarma et al., 2015; Solanki et al., 2019), and regulation of microbial communities (Zhang L.-N. et al., 2019). MSBCAs appear to have higher efficiency for control of soil-borne disease than SSBCAs (Figure 1A).

Synergistic and/or additive effects exerted by carefully selected microbial consortia might explain their superior efficacy compared to single SSBCAs. In simple cases, MSBCAs consist of only two strains, e.g., a fungus and a bacterium where one or both have biocontrol activities. A consortium consisting of *Trichoderma asperellum* GDFS1009 and *Bacillus amyloliquefaciens* ACCC1111060 was found to be more efficient against infection by *Botrytis cinerea* (the agent of gray mold disease) than the individual strains (Wu et al., 2018). Similarly, when *Trichoderma virens* GI006 was combined with *Bacillus velezensis* Bs006, efficiency against *Fusarium* wilt of cape gooseberry was enhanced (Izquierdo-García et al., 2020). A bacterial consortium of *Chitionophaga* sp. 94 and *Flavobacterium* sp. 98 conferred more consistent protection against the infection of root rot, the infection of sugar beets by *Rhizoctonia solani* than the individual community members (Carrión et al., 2019). Thus, MSBCAs are capable of providing more effective protection of the hosts than inoculation with single-strains. A model resident bacterial community composed of five non-virulent *Ralstonia* spp. strains was more efficient at reducing the spread of the bacterial wilt of tomato caused by *Ralstonia solanacearum* than the single non-virulent strains (Wei et al., 2015). Further examples documenting the superior action of MSBCAs against soil-borne plant pathogens are listed in Table 1.

The enhancement of disease inhibition by MSBCAs is widely thought to be due to the addition of different features for control (Pierson and Weller, 1994; Sarma et al., 2015). Occupation of distinct niches in the rhizosphere may avoid competition among probiotic microorganisms, resulting in more stable rhizosphere communities (Pierson and Weller, 1994; Pliego et al., 2008; Thomloudi et al., 2019). Modulation of genetic elements (Lutz et al., 2004) and suppression of a broader range of phytopathogens (Pierson and Weller, 1994; Thomloudi et al., 2019) may account for the elevated biocontrol activity in microbial communities. In addition, some key features related to the disease-controlling effect of BCAs, including rhizosphere colonization and suppression of pathogen growth, can be promoted in consortia via a complex network of microbe−microbe interactions. This interplay might serve as the selective force building plant-associated microbial communities (Hassani et al., 2018). Members of the MSBCAs apply interspecies communication as a strategy to improve their control of soil-borne diseases.

## Microbial Interactions Promote Rhizosphere Colonization

### Colonization in the Plant Rhizosphere

Efficient colonization of the rhizosphere is the first and fundamental step to protect plants from soil-borne pathogens by BCAs. Insufficient rhizosphere colonization can impair the beneficial effects of the biocontrol microbial strains, resulting in reduction or failure of disease control. Inoculation with MSBCAs may enhance the colonization of the rhizosphere by biocontrol microbes. The rhizoplane colonization ability of a five-strain bacterial consortium suppressing a sudden wilt disease of *Nicotiana attenuata* was enhanced compared to that of each single community member (Santhanam et al., 2019). Survival of *Pseudomonas* species communities inhibiting bacterial wilt disease of tomato increased with increasing diversity (Hu et al., 2016). In addition, the total bacterial abundance on bean root tips rose when a two-membered biocontrol *Pseudomonas* species consortium for anthracnose was added (Bardas et al., 2009). Thus, using disease-controlling microorganisms as multi-strain consortia can indeed promote rhizosphere colonization by BCAs (Figure 1B). Such positive effects on microbial colonization may be due to positive regulation of some colonization-related biological processes, such as biofilm formation, growth and migration, by the interactions among microorganisms within consortia.

A promising example of successful use of empirical mixtures of BCA is the combination of the fungus *Trichoderma* spp. and the biocontrol bacterium *Bacillus velezensis.* According to *in vitro* observations the microbes appear to be incompatible. *B. velezensis* FZB42 produces an arsenal of antifungal compounds. The lipopeptides bacillomycin D and fengycin act antagonistically against filamentous fungi (Chowdhury et al., 2015) and it is to be expected that the bacilli might inhibit growth of *Trichoderma* when applied together. However, supernatants of *B. velezensis* stimulated growth of *Trichoderma virens* under *in vitro* conditions. *Vice versa*, addition of *Trichoderma* conidia did not affect viability of *B. velezensis* suggesting high compatibility of both microbes. Adhesion of *B. velezensis* spores to the conidia of *T. virens* without affecting their morphology was observed, supporting compatibility of both soil inhabitants (Izquierdo-García et al., 2020).

Germination of fungal conidiospores and *Bacillus* endospores, is a critical step in successful colonization of BCAs. The contact of *Trichoderma* conidia with *Bacillus* biofilms did not impair the ability of fungal spores to germinate and establish the fungus in soil (Izquierdo-García et al., 2020). The mycelia of *Trichoderma* can serve as a supporting layer for formation of bacterial biofilms and can aid bacterial migration in the soil. Growth of bacteria was supported by nutrients present in the fungal exudates (Warmink et al., 2011; Triveni et al., 2012).

### Enhanced Biofilm Formation

Microbial colonization of plant roots can be promoted by the formation of biofilms (Fan et al., 2011; Beauregard et al., 2013). Biofilms are communities of surface-associated microorganisms encased in a self-produced extracellular matrix composed of exopolysaccharides, proteins and sometimes DNA (Vlamakis et al., 2013). Beside the well-studied single-species biofilms, rhizosphere microorganisms belonging to multiple taxa are able to form multi-species biofilms, of which the formation might be elevated by the microbe−microbe interactions within biofilm communities (Figure 1B). In a previous study, a consortium of five native bacterial isolates was found to be able to colonize the roots of *N. attenuata* by forming multiple-taxa biofilms on the root surfaces. Furthermore, under both *in vitro* and *in vivo* conditions, the amount of biofilm produced by each individual strain was significantly less than the biofilms formed by the five-membered bacterial community, which indicating a synergistic biofilm formation by the consortium (Santhanam et al., 2019). Similarly, a three-species biocontrol community composed of *Xanthomonas* sp. WCS2014-23, *Stenotrophomonas* sp. WCS2014-113 and *Microbacterium* sp. WCS2014-259 showed synergy, as the combination of three formed more biofilm than the single strains. Moreover, colonization of host roots by this community was stimulated by enhanced biofilm formation (Berendsen et al., 2018). Although the mechanisms of such positive effects on biofilm production are unclear, the improved efficacy can be attributed to the cooperative microbial interactions in consortia, triggering increased extracellular matrix deposition and cell-to-cell signaling (Santhanam et al., 2019).

### Syntrophic Microbial Growth Promotion

Colonization of the rhizosphere requires robust microbial growth, which can be greatly improved by syntrophy, a nutritional situation in which multiple microorganisms combine their metabolic abilities to catabolize a substrate that cannot be degraded by either one of them alone (Morris et al., 2013; Mee et al., 2014). For example, *Azospirillum brasilense*, a well-known plant growth-promoting rhizobacterium (PGPR), is not able to use certain sugars and polysaccharides as carbon sources for growth *in vitro*. However, it is capable of associating with sugar- or polysaccharide-degrading bacteria, establishing a metabolic association where the sugar- and/or polysaccharide-degrading bacteria degrade the metabolites to products, which can be utilized as carbon source by *A. brasilense*. In turn *A. brasilense* provides the sugar- or polysaccharide-degrading bacteria with nitrogen by fixing the atmospheric N_2_ (Bashan and Holguin, 1997; Bashan, 1998). Such a synergistic catabolic effect on sugars and polysaccharides may boost the survival of *A. brasilense* in the rhizosphere, because plant roots release 5−21% of their photosynthetically fixed carbon as root exudates (Bais et al., 2006; Huang et al., 2014), including sugars and polysaccharides, which are used by the rhizosphere microbial communities. Beside nutrients, there are always microbial growth-inhibiting materials in the rhizosphere. While methanol can suppress the growth of methanotrophs in the rhizosphere, the methanotrophs are able to survive by coexisting with *Hyphomicrobium* spp. to build a rhizospheric microbial association, in which the *Hyphomicrobium* spp. is capable of removing methanol (Liechty et al., 2020). Therefore, the promotion of the growth of rhizosphere microbes can be achieved by syntrophic interactions leading to effective nutrient utilization and removal of harmful substances (Figure 1B).

### Facilitated Migration

Another crucial microbial trait for rhizosphere colonization is motility, defined as the ability of microorganisms to move or to perform mechanical work at the expense of metabolic energy (Harshey, 2003). There are six different categories of surface motility including swimming, swarming, gliding, twitching, sliding and darting (Harshey, 2003). According to Allard-Massicotte et al. (2016), motility is required for early root colonization by BCAs. The migration of microorganisms can be enhanced by the interactions among community members. For example, fungal hyphae are capable of serving as vectors for the dispersion of bacteria in the rhizosphere, which is known as a “fungal highway” (Kohlmeier et al., 2005; Warmink et al., 2011; Figure 1B). In a recent study, Zhang et al. (2020) showed that rhizobia use mycelia of *Phomopsis liquidambaris* as dispersal networks to migrate into legume rhizospheres and to trigger nodulation. Extraradical mycelium formed by the mycorrhiza fungus *Glomus formosanum* CNPAB020 can facilitate the translocation of *Bradyrhizobium diazoefficiens* USDA 110 in the rhizosphere (de Novais et al., 2020) in addition to its main activity in nutrient transfer. Prokaryotic cells are able to facilitate dispersal of non-motile asexual fungal spores as well (Figure 1B). Conidia of *Aspergillus fumigatus*, a non-motile rhizosphere fungus, can be transported by the rhizobacterium *Paenibacillus vortex* from niches of adverse growth conditions. Fungal mycelia may act as bridges to allow *P. vortex* to cross air gaps, which can be mutually facilitated dispersal, benefiting the life cycles of both of these very different rhizosphere inhabitants (Ingham et al., 2011). The enhanced dispersal may also occur between distinct bacterial species. An ampicillin-sensitive *P. vortex* strain was capable of swarming and colonizing on ampicillin plates using non-motile ampicillin-resistant *Escherichia coli* as cargo, dispersing both bacteria (Finkelshtein et al., 2015; Venieraki et al., 2016). Co-swarming or transporting other bacterial species may expand the abilities of the partners in occupying and exploiting ecological niches in diverse environments including the rhizosphere (Venieraki et al., 2016). Hence, interactions among the microbial components of a given community may bring about facilitated microbial migration, essential for efficient rhizosphere colonization.

In brief, microbe−microbe interactions can play a positive role in promoting rhizosphere colonization by beneficial microorganisms through boosting biofilm formation, microbial growth, migration inside of the microbiome, and interacting with plant roots. Thus, utilization of MSBCAs performing active interactions among their members may improve survival of disease-suppressing microbes, and their adaption to complex and changeable environmental conditions. In consequence, they may be able to stabilize their beneficial effects for the inhibition of soil-borne diseases.

## Microbial Interactions Enhance Growth Suppression of Soil-Borne Pathogens

Multi-strain biological control agents are able to exhibit stronger suppressive efficacy on the growth of soil-borne pathogens than SSBCAs. For instance, a bacterial strain mixture involving *Bacillus subtilis* S2BC-1 and GIBC-Jamog showed greater anti-fungal activity against the tomato vascular wilt pathogen, *Fusarium oxysporum* f.sp. *lycopersici*, than each of the individual strains (Shanmugam and Kanoujia, 2011). Similarly, *Pseudomonas fluorescens* T5 showed no inhibition against *Rhizoctonia solani in vitro*. However, when it was applied together with four non-antagonistic bacterial strains isolated from the rhizosphere of *Tamarindus*, this five-species bacterial community exhibited strong suppression of growth of *R. solani* (Kannan and Sureendar, 2009). Although the understanding of the enhanced pathogen-inhibiting effect of biocontrol consortia is limited, changes in resource competition and secretion of antimicrobial compounds triggered by microbial interactions may contribute to the enhanced suppression (Figure 1B).

### Boosted Competition for Resources

Resource competition is a basic mechanism by which BCAs may protect plants from pathogens, implying that the beneficial microorganisms are able to rapidly and efficiently utilize the limited resources in the vicinity of the plant hosts to restrict or suppress the growth of phytopathogens. Plant exudates on root surfaces and in their surrounding rhizosphere, are the primary sources of nutrients for the rhizosphere microbiome. Successful suppression depends on the competition for nutrients in root exudates by biocontrol microbes and soil-borne pathogens. This contest can be elevated by the microbial interplay inside MSBCAs (Figure 1B). Two biocontrol consortia for tomato bacterial wilt caused by *R. solanacearum*, consisting of eight *Pseudomonas* and five non-virulent *Ralstonia* strains, exhibited much stronger inhibiting effects on the population density of *R. solanacearum* than each individual strain. The enhanced inhibition is caused by an increase of niche overlaps exerted by these consortia with *R. solanacearum.* Niche overlaps may be defined as ‘likeness’ between the communities and *R. solanacearum* in the catabolism of 48 different single-carbon resources found in tomato root exudates (Wei et al., 2015; Hu et al., 2016). The more diverse soil bacterial communities are, the better they are able to acquire many of the 31 individual carbon sources typical for soil, than the pathogen *E. coli* O157:H7 (van Elsas et al., 2012). Limited assimilatable iron resources remain in the rhizosphere, following the competition between disease-suppressing microorganisms and soil-borne pathogens (Gu et al., 2020). Many soil microbes scavenge iron by secreting siderophores, a chemically diverse group of secondary metabolites with a high affinity for iron, because iron predominantly occurs in soil in its insoluble ferric Fe (III) form (Traxler et al., 2013; Traxler and Kolter, 2015). The siderophores can both, to facilitate and suppress competitors, depending on whether the competitors possess the transporters or channels for siderophore uptake. The production of siderophores can be positively regulated by interspecies interactions among soil microbes. The interplay of *Streptomyces coelicolor* with five other soil actinobacteria increased the diversity of siderophores. Production of desferrioxamines by *S. coelicolor*, was triggered by siderophores from neighboring strains (Traxler et al., 2013). Therefore, the disease-inhibiting microorganisms in the rhizosphere may acquire elevated capability to utilize resources through microbial associations (Figure 1B).

### Stimulated Synthesis of Antimicrobial Compounds

Microorganisms are able to synthesize a multitude of compounds with antimicrobial activity, which is an important mode of action for direct inhibition or lethality on the microbial opponents in environments. So far, there have been a large number of reports of the antimicrobials produced by BCAs exhibiting suppressing effects on the growth of phytopathogens. These studies mainly focus on the biocontrol strains from the genera *Bacillus*, *Pseudomonas*, and *Trichoderma*, well known for the production of antibiotics including lipopeptides, polyketides, bacteriocins, phenazines, 2,4-diacetylphloroglucinol (DAPG) and chitinase (Ghisalberti and Sivasithamparam, 1991; Haas and Keel, 2003; Chen et al., 2007). Some metabolites with inhibitory functions are found in low concentration or are not expressed in pure culture but may be upregulated in a community (Nützmann et al., 2011; Brakhage, 2013; Pishchany et al., 2018). Lutz et al. (2004) examined the molecular interactions between bacterial and fungal BCAs, the DAPG-producing *P. fluorescens* and chitinase-producing *Trichoderma atroviride* P1. DAPG enhanced the expression of the *nag1* chitinase gene, indicating that the positive regulation of key biocontrol genes may take place while mixing antagonists. Co-culturing the endophytic fungus *Fusarium tricinctum* with *Bacillus subtilis*, resulted in as much as a 78-fold increase in the accumulation of secondary metabolites including compounds with antimicrobial efficacy (Ola et al., 2013). Therefore, specific interactions among microorganisms belonging to different domains may enhance production of antimicrobial compounds. Not only are the microbial interactions able to upregulate the production of known antimicrobial compounds, but interactions may also activate the biosynthesis of hitherto unknown compounds with antimicrobial activity (Figure 1B). A novel antibiotic named amycomicin has been recently described (Pishchany et al., 2018). The production of this compound is dependent on the interaction between two soil-dwelling actinobacteria, *Amycolatopsis* sp. AA4 is the producer strain and *Streptomyces coelicolor* M145 is an inducer. According to these examples the synthesis of antimicrobial compounds can be stimulated or activated through both, inter- and intra-domain microbial interactions.

Therefore, the modulating effect of microbial interactions on resource competition and production of antimicrobial compounds may contribute to strengthening the inhibition of growth of pathogens (Figure 1B). Thus, applying BCAs as multi-strain mixtures can elevate the ability of biocontrol microorganisms to compete for the resources needed for rhizosphere survival with soil-borne pathogens and to stimulate the production of compounds toxic to specific pathogens. The increased niche overlaps and biosynthesis of novel antimicrobial compounds induced by microbe−microbe interactions may facilitate the BCAs to suppress a broader range of phytopathogens. The positive impact of interactions within MSBCAs may result in more efficient growth suppression of soil-borne pathogens, and improve the efficiency of soil-borne disease control by disease-inhibiting microbes.

## Interactions of Microbial Communities With Plants and Soil

Plants rely on rhizosphere microbiota to facilitate nutrient acquisition, in exchange for carbon-rich root exudates for bacterial nutrition. In addition, the rhizosphere microbiome is important for plant health and fitness (van der Heijden et al., 2008). The plant root microbiome consists of prokaryotic bacteria, eukaryotic filamentous fungi, and oomycetes. Besides a core microbiome ubiquitous in a multitude of hosts and geographical regions, a variable part of the microbiome is shaped by secretion of species-dependent plant secondary metabolites, which belong to diverse classes, such as coumarins, benzoxazinoids, phytoalexins and triterpenes (Jacoby et al., 2020). Consequently, diversity of species along the bulk-soil to root microbiota was found gradually decreasing. Positive correlations dominate within each of the three kingdoms. Reconstitution experiments performed with synthetic mono- or multi-kingdom microbial consortia and germ-free *Arabidopsis* plantlets revealed that the bacterial microbiota protects plants against potentially pathogenic fungi and oomycetes by mainly negative factors exerted against filamentous fungi (Durán et al., 2018). Widely distributed members of the core microbiota such as *Variovorax*, a gram-negative beta-proteobacterium, and *Pseudomonas* appeared to be important for plant protection but individual members of other bacterial taxa could overtake their function in biocontrol. Therefore, addition of an SSBCA or MSBCA might have positive effects in complex systems of agriculture and forestry.

The plant immune system also affects the composition of the microbiota in the vicinity of plant roots. The root-microbiome may expand plant immunity and acts as an additional layer of defense against plant pathogens (Teixeira et al., 2019). Interaction of beneficial microbes with plant roots can result in systemic host resistance to pathogens, which may be due to the activation of induced systemic resistance (ISR) (Sarma et al., 2015). In addition to promoting rhizosphere colonization and suppressing soil-borne pathogen growth, inducing enhanced plant defense responses to pathogens has been described in many studies as another important feature employed by the MSBCAs for their elevated disease-controlling effect. The additive or synergistic efficacy of the biocontrol consortia on the induction of elevated host immunities to plant pathogens is directed by activating several distinctive metabolic and signaling pathways against a given pathogen (Jain et al., 2012; Alizadeh et al., 2013; Sarma et al., 2015). However, how interactions among the members of MSBCAs can effectively boost specific systemic resistance to soil-borne pathogens remains to be better illustrated. One possible hypothesis is that the microbe-microbe interplay within the biocontrol consortia might lead to the production of larger amounts of specific elicitors and potent compounds capable of more efficiently eliciting ISR (Figure 1B).

Many root-associated gram-positive and gram-negative bacteria are able to produce plant growth hormones, such as indole-3-acetic acid (IAA) and thus promoting plant root growth, when auxin production does not exceed a critical level (Vessey, 2003; Lugtenberg and Kamilova, 2009). In case of some pathogenic bacteria, IAA production exceeds the critical threshold needed for plant growth and may negatively affect plant health (Spaepen et al., 2007; Subramoni et al., 2014; Segev et al., 2016). Some beneficial root-associated microbes such as *Variovorax* possess the IAA catabolic gene cluster and can reverse root growth inhibition occurring at high IAA concentrations by degrading IAA (Fitzpatrick et al., 2020).

Soil not only supports plant and animal life, but also hosts myriad microorganisms inside, referred to collectively as the soil microbiome (Berg and Smalla, 2009; Fierer, 2017; Jansson and Hofmockel, 2018; Thakur and Geisen, 2019), which governs biogeochemical cycling of macronutrients, micronutrients and other elements vital for the growth of plants and animals (Jansson and Hofmockel, 2020). The interactions between microbes and soil have always drawn the attention of microbiologists and ecologists. It has been widely accepted that microbial communities inhabiting soil are capable of alternating its physicochemical properties by organic litter deposition and metabolic activities (Jacoby et al., 2017; Jansson and Hofmockel, 2020), for example, by improving water retention (Naylor and Coleman-Derr, 2017), increasing carbon storage (Jansson et al., 2018) and mineral nutrition contents (van der Heijden et al., 2008; Jacoby et al., 2017). Vice versa, the variability in soil traits may impact the composition and function of soil microbial communities (Peiffer et al., 2013; Yang et al., 2019; Chen et al., 2020; Wang et al., 2020). Our increasing awareness of the influences of soil-feature changes on the microbiome has resulted in an emerging urgency to elevate the suppressing effect of soil microbiota against phytopathogens by managing the soil properties. Wang et al. (2020) demonstrated that the addition of biochar to the soil not only raises the pH and the available nutrient content, but also augments fungal richness and diversity, especially the abundance of potential biocontrol fungi, which led to the inhibition of *Phytophthora* blight of pepper. Similarly, biochar amendment controlled bacterial wilt through changing soil chemistry and the composition of the microbial community. The application of biochar specifically enriched beneficial bacteria and decreased pathogen abundance (Chen et al., 2020). Furthermore, Yang et al. (2019) showed that wheat straw return significantly increased soil nitrogen and reduced the relative abundance of pathogenic fungal genera in the soil microbial community, indicating a potential for disease control. Thus, promoting the biocontrol effects of the soil microbial community against soil-dwelling pathogens by manipulating soil features is a promising strategy for soil-borne disease management. Moreover, understanding the interplay between the soil and its associated microbiota will expand our knowledge about the impact of abiotic factors on biological soil-borne pathogen control.

## Microbial Interactions and Construction of MSBCAs

Application of BCAs in the community context as MSBCAs can increase the ability to control soil-borne diseases of crops through interaction-mediated promotion of rhizosphere colonization outcompeting soil-borne pathogens. Thus, construction and utilization of MSBCAs could augment soil-borne disease control in sustainable agriculture and forestry. So far, there are at least two strategies for preparing effective MSBCAs, (i) mixing the existing SSBCAs according to empirical criteria, and (ii) assembling MSBCAs by applying the reductionist SynCom approach, also named RSC (Liu et al., 2019). Using either one of the two strategies, microbe-microbe interactions need to be taken into account.

### Mixing the Compatible and Diverse SSBCAs According to Empirical Criteria

Combining beneficial microbial isolates that may enhance the effect achieved by single isolates dates back to the discovery of PGPR (Kloepper et al., 1980). Selecting proper strains is critical. We noted that microorganisms used for developing biocontrol consortia were often selected according to their individual disease suppressive capacity. However, except for this property, no precise selection standards have been adopted to choose microbial components (Sarma et al., 2015; Thomloudi et al., 2019). This approach often results in equal or even lower efficacy of the multi-strain mixtures compared to the individual strains (Sarma et al., 2015). Thus, it is necessary to carefully evaluate the compatibility and interactions of the candidate strains before the MSBCA consortium is established. We propose, in addition to the disease-inhibiting activity of individual strains, to consider two interaction-related properties, (i) compatibility, and (ii) diversity.

The members of a probiotic consortium are considered to be compatible when they do not inhibit growth of each other during their *in vitro* co-culture and/or in rhizosphere colonization competition assays (Liu et al., 2018; Thomloudi et al., 2019). Co-inoculation with incompatible isolates might hinder one or more microbial agents from reaching the appropriate population threshold for plant disease control (Haas and Defago, 2005). The results of the *in vitro* co-culture compatibility tests often represent the interactions occurring among the members of the consortium. However, variation in media used to test *in vitro* compatibility (Lyons and Kolter, 2017), the colonization of different ecological niches on roots (Pliego et al., 2008), and interference among mechanisms for disease control (Stockwell et al., 2011) can lead to inconsistent compatibility assays. Thus, compatibility among members of a synthetic microbial community should be considered as a prerequisite in the engineering of MSBCAs applied to plants, and should be verified by further assays.

In addition, the degree of microbial diversity affects the assembly, survival, and functionality of BCAs in the rhizosphere and their ability to inhibit soil-borne diseases (Hu et al., 2016). First, a high level of species diversity can increase the resources that microbial species can collectively use as a community (the niche breadth), and enable microorganisms to survive in the rhizosphere more efficiently (Wei et al., 2015). Second, the amount and number of secondary metabolites that suppress pathogen growth increase with increasing taxonomic diversity in MSBCAs (Raaijmakers and Weller, 1998; Jousset et al., 2014). A combination of different secondary metabolites produced jointly by diverse microbes may strengthen the antagonistic effect against pathogens (Loper et al., 2012). Therefore, MSBCAs of high diversity could be more adaptive to the pressure of rhizosphere environments and act more efficiently against soil-borne plant diseases.

**FIGURE 2:**
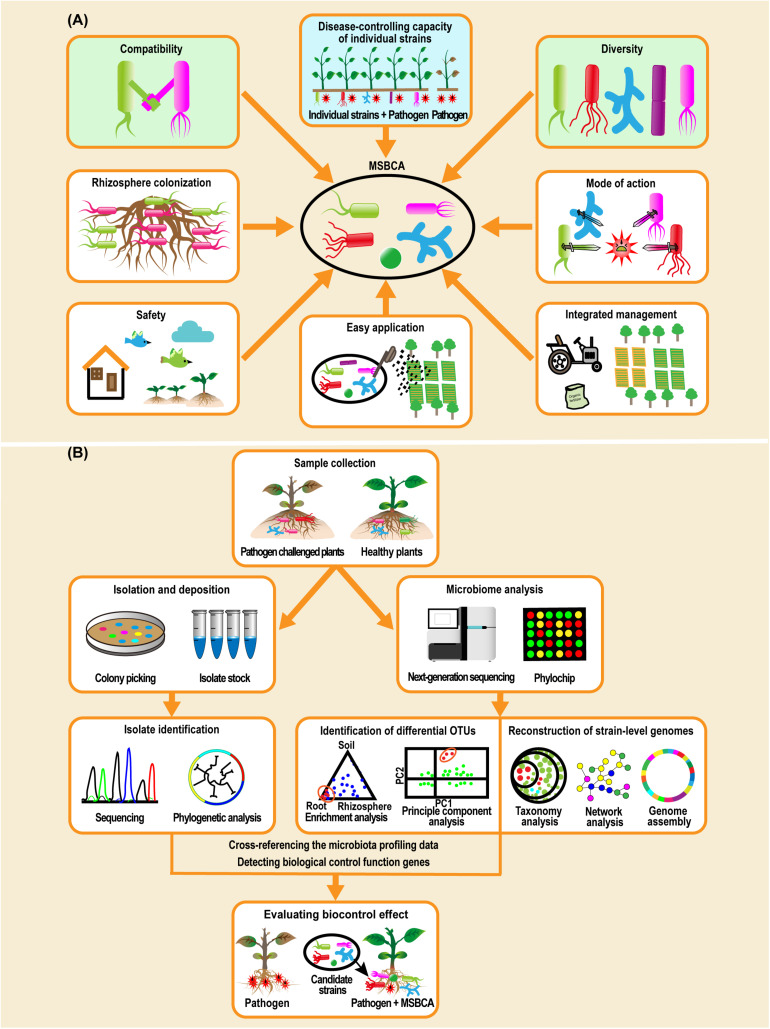
Biological features need to be considered when constructing multiple-strain biological control agents (MSBCAs) and workflow of assembling MSBCAs using a reductionist synthetic community (Vorholt et al., 2017; Liu et al., 2019). **(A)** Biological features involving disease-suppressive effects of each individual strain, compatibility, diversity, microbial colonization of rhizosphere, mode of action for disease control, safety to humans and the environment, easy application and convenience to be incorporated into an existing management system (Raupach and Kloepper, 1998; Sikora et al., 2010; Bashan et al., 2013; Grosskopf and Soyer, 2014; Ahkami et al., 2017), need to be taken into account when establishing the MSBCAs for soil-borne diseases. **(B)** In general, methodology of constructing MSBCAs by a reductionist synthetic community approach is built on the conception of host-mediated selection of plant-associated microbiota (Mueller and Sachs, 2015). Microbiome analysis by 16S rRNA gene amplicon sequencing or metagenomics sequencing, or by PhyloChip analysis, in parallel to the extended microbial strain isolation to achieve as much diversity as possible, is an early step to pick the potential disease-suppressing species by identifying the differential OTUs between the microbiome of the samples collected from pathogen challenged and control plants (Berendsen et al., 2018), or by reconstructing strain-level genomes based on functional diversity (Carrión et al., 2019). Then, after cross-referencing the microbiota profiling data with the taxonomic identities of the isolates in comprehensive culture collections (Niu et al., 2017; Berendsen et al., 2018), or by detecting the genes encoding the functions of biological control in the genomes of cultivated isolates (Carrión et al., 2019), the candidate strains will be characterized and selected for the multi-strain community, of which the disease-reducing effects will be further evaluated.

In summary, compatibility and diversity are two interaction relevant factors (Figure 2A) that may determine the success of MSBCAs. Some additional traits, such as microbial colonization of the rhizosphere, mode of action for disease control, safety to humans and the environment, ease of application and convenience of management systems need to be considered, when establishing the biocontrol microbial communities for soil-borne diseases (Raupach and Kloepper, 1998; Sikora et al., 2010; Bashan et al., 2013; Grosskopf and Soyer, 2014; Ahkami et al., 2017).

### Building MSBCAs by the Reductionist SynCom Approach

Although empirically combining existing microbial isolates with biocontrol activity is useful, it is nearly impossible to predict efficiency of such consortia in suppressing plant disease and strengthening plant growth in the context of the whole plant microbiome. In contrast, utilizing a reduced number of representative members of the target host microbiota to build SynCom (Vorholt et al., 2017; Liu et al., 2019) will likely simplify handling and production of such MSBCAs. SynCom analysis performed in gnotobiotic systems allows us to study the effect of the plant microbiota on host fitness under different environmental circumstances. It also allows us to investigate microbe−microbe interactions and microbial gene functions (Carlström et al., 2019; Liu et al., 2019), and to construct novel MSBCAs.

Several microbial communities able to suppress plant diseases have been assembled via the reductionist SynCom approach based on microbiome analysis and comprehensive culture collections (Liu et al., 2019). A synthetic bacterial consortium was constructed, able to reduce the severity of the maize seedling blight caused by *Fusarium verticillioides* (Niu et al., 2017). The biocontrol effect of the synthetic community against *F. verticillioides* was stronger than that of each individual strain. To prepare this synthetic community, Niu et al. (2017) started from microbiota established by maize roots, which were identified by 16S rRNA gene amplicon sequencing and additional strain cultivating methods. A greatly simplified SynCom was obtained, consisting of seven strains, *Enterobacter ludwigii*, *Stenotrophomonas maltophilia*, *Ochrobactrum pituitosum*, *Herbaspirillum frisingense*, *Pseudomonas putida*, *Curtobacterium pusillum*, and *Chryseobacterium indologenes*, representing three of the four most dominant phyla found in maize roots.

A three-membered bacterial community able to induce systemic resistance in *Arabidopsis thaliana* against *Hyaloperonospora arabidopsidis* (downy mildew) was constructed (Berendsen et al., 2018) via host-mediated microbiome selection (Mueller and Sachs, 2015). Carrión et al. (2019) showed that infection of sugar beets by a fungal pathogen, *Rhizoctonia solani*, is hindered by an endosymbiotic community of bacteria living inside plant roots. This endophytic community was enriched for Chitinophagaceae and Flavobacteriaceae harboring chitinase genes and biosynthetic gene clusters encoding non-ribosomal peptide synthetases and polyketide synthases. A MSBCA consortium of *Chitinophaga* and *Flavobacterium* strains was established, which consistently suppressed fungal root disease. Carrión et al. (2019) concluded that endophytic root microbiomes may harbor many functional traits that can protect synergistically their host plants (Carrión et al., 2019).

## Conclusion and Future Perspectives

Building MSBCAs by a reductionist SynCom approach (Figure 2B) offers the chance to accurately and rapidly pick out the microbial strains qualified for establishing the MSBCA from thousands of isolates found in the natural host microbiome. In this way, the crucial disease control-interactions present in the plant microbiome (Hassani et al., 2018) can be mirrored in the few selected strains used for the MSBCA. Establishing SynComs should be the method of choice. SynComs represent a helpful complement to pesticides, and might be combined in future application with effective empirical mixtures and/or single representatives of existing SSBCAs.

Utilization of selected beneficial microorganisms in the community is an effective approach to improve the efficiency of BCA (Figure 1A; Sarma et al., 2015; Mazzola and Freilich, 2017; Vorholt et al., 2017; Woo and Pepe, 2018). A necessary precondition for its success is the analysis of the microbial interactions among the members and the effect exerted by the MSBCA on plant health (Figure 1). When designing a MSBCA, two crucial interaction-related factors, compatibility and diversity, need to be considered (Figure 2A). Constructing MSBCA by combining microbes with great taxonomic distance appears desirable. We recommend a reductionist SynCom approach based on the principle of host-mediated microbiome selection (Mueller and Sachs, 2015), and selection of representative microbes to form efficient biocontrol consortia. This allows us to assemble customized MSBCAs depending on the specific requirements of disease management in different crops and environments. This strategy will result in protecting against distinct pathogens and might be comparable to the concept of “precision medicine” for human health (Berg et al., 2020), that advocates treatments of patients on a personalized level (Collins and Varmus, 2015) based on the patient’s genome sequence and their specific genome-environment interaction. Beside the practical use of MSBCAs as biopesticides, they may also serve as useful tools for investigating how microbial interspecies interactions affect plant microbiome assembly (Niu et al., 2017), and how evolutionary processes act on the plant holobiont (integrating the plant, the microbiome and the environment) (Hassani et al., 2018).

In this review, we summarize the potential mechanisms deployed by microbial components of communities to improve their disease-suppressing functions. Our understanding of these processes at the level of molecular mechanisms is rudimentary, especially the mechanisms of the initiation of rhizosphere colonization and the resulting elevated host immunity. Next, the technology of functional genomics, transcriptomics, proteomics and metabolomics will need to be applied to elucidate the genetic basis of enhanced biofilm formation, syntrophic microbial growth promotion and migration, and enhanced ISR. Although a 16S rRNA gene amplicon sequencing-based reductionist SynCom approach is useful to characterize MSBCAs, the relatively short reads may not achieve the taxonomic resolution needed to distinguish related strains (Edgar, 2018; Fuks et al., 2018). Thus, beside the high cost of a metagenomics approach, the utilization of modified 16S rRNA gene amplicon sequencing-based methods with improved resolution, such as full-length 16S rRNA gene amplicon sequencing (Callahan et al., 2019), may be expanded in the future when constructing the SynComs with biocontrol activity. In addition to the disease-suppressing function of the SynComs, their plant growth-promoting effects are worth further investigation (Zhang J. et al., 2019; Zhuang et al., 2020). So far, most MSBCAs have been applied in agriculture (Table 1), Using BCAs in forestry for plant disease control should be recommended. Finally, as agrochemical companies such as BASF, Syngenta and Bayer have developed and launched several MSBCA-based commercialized products for sustainable management of soil-borne pathogens, application of MSBCAs should bring more efficient control of soil-borne diseases in agriculture, horticulture, and forestry.

## Author Contributions

BN and RB conceived the idea, designed the outlines of the review, and wrote the manuscript. WW and ZY prepared the figures and the table. VC, RS, and HS revised the manuscript. All authors listed have made a substantial, direct and intellectual contribution to the work, and approved it for publication.

## Conflict of Interest

The authors declare that the research was conducted in the absence of any commercial or financial relationships that could be construed as a potential conflict of interest.
